# Neutrophil Migration and Adhesion Molecule Expression after Acute High-Intensity Street Dance Exercise

**DOI:** 10.1155/2018/1684013

**Published:** 2018-07-08

**Authors:** Leandro Borges, Alexandre Dermargos, Stuart Gray, Maysa B. Barros Silva, Vinicius Santos, Tania Cristina Pithon-Curi, Renata Gorjão, Elaine Hatanaka

**Affiliations:** ^1^Institute of Physical Activity and Sport Science (ICAFE), Cruzeiro do Sul University, São Paulo, SP, Brazil; ^2^Universidade Paulista (UNIP), São Paulo, SP, Brazil; ^3^Institute of Cardiovascular & Medical Sciences, University of Glasgow, Glasgow, UK

## Abstract

The physical demands of street dancing may result in inflammation and changes in leukocyte numbers/function, impairing the health of dancers. Herein, we investigated the effect of street dancing on inflammation, adhesion molecules, and neutrophil function. Fifteen amateur dancers (mean ± SE: age 22.4 ± 1.08 years, BMI 24.8 ± 0.69 kg/m^2^, and body fat 12.3 ± 1.52%) participated in a single high-intensity street dance class. Blood samples were taken before and after the class. The dance class had no effect on the plasma concentration of CRP, TNF-*α*, IL-6, IL-10, and IL-8; however, we noted an increase in levels of IL-1*β* (4.06%) and sL-selectin (17.67%). The dance class resulted in a 12.36% increase in neutrophil counts, while neutrophil CD62L expression and migration were reduced (25.27% and 78.92%, resp.). After the dance class, neutrophil production of IL-8 and TNF-*α* increased, respectively, by 59.75% and 49.23%, in the control condition, and 43.55% and 32.22%, after LPS stimulation. A single bout of street dancing induced inflammation and reduced neutrophil migration and adhesion molecule expression. These findings may contribute to a better understanding of the susceptibility to infection after acute dance exercise.

## 1. Introduction

Hip-hop dance, a term that encompasses many styles, including popping, breakdance, funk, house, krumping, voguing, and street dance [1], has grown in popularity with an increasing number of television shows and movies and attracted a great number of followers [2]. As with other such exercises, dancing improves motor control, attention, cognition, and physical fitness, but intense training frequently leads to acute and/or chronic injury [3, 4]. Indeed, a temporary suppression of the immune system, inflammation, and muscle damage after an acute bout of intense/exhaustive exercise are common findings. Increasingly, dancers are recognized as elite athletes and the physical demands placed on choreography and heavy performance loads can result in muscle damage and inflammation [5].

Under normal conditions, after muscle damage is induced, the inflammatory response is initiated by the release of inflammatory mediators, such as proinflammatory cytokines (i.e., tumor necrosis factor-*α* (TNF-*α*) and interleukin 1*β* (IL-1*β*)) into the injured tissue [6, 7]. When these events occur in an uncontrolled manner, the ensuing systemic inflammation can alter neutrophil numbers and activation status, which may lead to increased susceptibility to invasive microorganisms and may negatively affect the dancers' performance and health. Sequentially, the soluble forms of sL-selectin and CD62L (bound to the membranes of leukocytes), as well as other adhesion molecules, initiate the emigration of leukocytes, by regulating the flux of rolling neutrophils and inflammatory monocytes [8], playing a critical role for the initiation of leukocyte responses [9].

In order to understand inflammatory modulation by neutrophils, studies have used treatment with lipopolysaccharide (LPS) stimulus to induce changes in Toll-like receptor 4 signaling pathways and NF-kappa B activation, simulating the exaggerated host response to infection through the release of cytokines and adhesion molecules [6]. Moreover, one of the first steps in infection control is the coordinated signaling that orchestrates the migration of neutrophils to the inflammatory site [10]. Herein, we investigated the effects of a single street dance class on markers of inflammation and immune function in dancers. Inflammation was monitored by plasma cytokines (TNF-*α*, interleukin-6 (IL-6), interleukin-10 (IL-10), and interleukin-8 (IL-8)) and C-reactive protein (CRP) measurement. We also assessed sL-selectin levels, neutrophil numbers, migration, expression of CD62L and IL-8, and TNF-*α* release by stimulated and nonstimulated neutrophils.

## 2. Methods

### 2.1. Subjects

With the approval of the Ethics Committee at the Cruzeiro do Sul University (0522013), 15 male dancers volunteered to participate in the study. All dancers signed an informed consent form agreeing to submit to the procedures involved in the study. The group had the following characteristics (mean ± standard error (SE)): age 22.4 ± 1.08 years, weight 70.8 ± 1.93 kg, height 169 ± 0.02 cm, body mass index (BMI) 24.8 ± 0.69 kg/m^2^, fat-free mass 61.5 ± 1.64 kg, body fat 12.3 ± 1.52%, and dance experience of 7 ± 13.78 years. The dancers had a training/performance history of 3.7 ± 0.39 hours a day, four days a week, and rested on weekends.

Participants were excluded from the study if they had a history of recent infection (last 15 days prior to the study), viruses, chronic lesions, diabetes, rheumatoid arthritis, hormonal dysfunction, lupus, or other inflammatory and hematology diseases (such as hemoglobinopathies) and took medication. To verify the dancer's health level, hematological parameters were assessed. The group presented the following characteristics (mean ± SE): red blood cells (RBC) 5.4 ± 0.14 × 10^3^/mm^3^, white blood cells (WBC) 9 ± 0.53 × 10^3^/mm^3^, hemoglobin (Hb) 14.7 ± 0.29 g/dL, hematocrit (Ht) 44.2 ± 0.88%, mean red cell volume 83 ± 1.86 fL, and hemoglobin concentration per red blood cell (MCHC) 33.3 ± 2.40%.

### 2.2. Study Design and Blood Collection

For this study, dancers were asked to rest for 72 hours before the street dance class. During the class, all participants had their heart rate (HR) monitored using *Polar* HR monitors and danced for 60 minutes at high intensity. This was defined according to the following intensities via HR reserve: at 85 ± 5.9% in the first 20 minutes, at 88.9 ± 3.9% between 20 and 40 minutes, and at 89.2 ± 3.3% between 40 and 60 minutes of the class.

Twenty milliliters (mL) of venous blood was collected before and immediately after the street dance class. Blood samples were drawn into BD Vacutainer® tubes, containing heparin. After collecting the samples, blood was centrifuged at 400 ×g for 10 minutes and the plasma was used to evaluate cytokines and CRP. Moreover, neutrophils were isolated from whole blood for cellular analyzes.

### 2.3. CRP and Cytokine Measurements

Plasma CRP and cytokines (TNF-*α*, IL-6, IL-10, IL-8, sL-selectin, and IL-1*β*) were measured by ELISA, according to the manufacturer's instructions (DuoSet Kit: Quantikine, R&D System, Minneapolis, MN, USA). A standard curve was produced for each set of samples, and the cytokine was assayed [11].

### 2.4. Isolation of Neutrophils

Cell isolation was carried out within 1 hour of venipuncture [12]. Human neutrophils were extracted from whole blood under endotoxin-free state using Histopaque®-1077 (Sigma Chemical Co., St. Louis, MO) following to the supplier's instructions and previously described by Böyum [13].

### 2.5. Cell Culture

After isolation, the cultured cell concentration obtained was 87.6 × 10^6^. Neutrophils (2.5 × 10^6^) were suspended in Roswell Park Memorial Institute (RPMI) 1640 medium supplemented with 0.3 g/L glutamine, 2.32 g/L Hepes, 2 g/L sodium bicarbonate, 100 *μ*g/mL streptomycin, 100 UI/mL penicillin, and 10% fetal bovine serum. The cells were then counted in a Neubauer chamber and immediately cultured at 37°C and 5% CO_2_, with and without LPS (5 *μ*g/mL) supplied by Sigma Chemical Co. (St. Louis, MO). After 18 hours of cell culture, the supernatant was collected and stored at ≤−80°C prior to cytokine analysis (IL-8 and TNF-*α*) by ELISA (Quantikine, R&D System, Minneapolis, MN, USA).

### 2.6. Expression of CD62L

The expression of CD62L (BD Biosciences, NJ, USA) was measured with a FACSAria II flow cytometer (Becton Dickinson, San Juan, CA, USA) from neutrophils (2.5 × 10^6^ cells/mL). In summary, the MEL-14 monoclonal antibody is conjugated with the fluorochrome fluorescein isothiocyanate (FITC) and recognizes an epitope localized to the lectin domain and CD62L binds a number of fucosylated, glycosylated, sulfated sialylated glycoproteins including CD34, MAdCam-1, and glycam-1. Data were acquired and analyzed using Diva software (Becton Dickinson), and results were expressed as the mean of the FITC fluorescence intensity. Ten thousand events were analyzed per experiment.

### 2.7. Neutrophil Chemotaxis

Neutrophil chemotactic responses were measured using 96-well disposable chemotactic plates (Neuro Probe), according to the manufacturer's instructions. Briefly, flat-bottomed chambers were filled with the chemotactic agent N-formyl-methionyl-leucyl-phenylalanine (fMLP) (Sigma Chemical, St. Louis, MO) (10 nmol/L) in phosphate-buffered saline (PBS) containing 0.01% albumin. Chemotactic membranes with a pore size of 5 *μ*m were fixed to the filter seat. The chamber assembly with neutrophils (2.5 × 10^4^ cells/mL) was incubated in a humidified 5% CO_2_ atmosphere at 37°C for 60 minutes. After incubation, the chamber was disassembled and the cells that migrated, crossing the plasma membrane, were counted directly in the Neubauer chamber and chemotactic responses were defined as the mean number of migrated neutrophils. The chemotactic factor fMLP (10 nmol/L) was used as a positive control for migration. Results were normalized to the number of cells that transmigrated under control conditions (PBS without fMLP).

### 2.8. Statistical Analysis

The results are expressed as the means ± SE. Statistical analysis of plasma cytokines and CRP, neutrophil counts, cell migration, and CD62L expression data was performed by paired *t*-test. Analysis of cytokine production by neutrophils (IL-8 and TNF-*α*) was performed using a one-way analysis of variance (ANOVA) accompanied by post hoc test (Student-Newman-Keuls multiple comparison) (InStat; GraphPad Software, San Diego, CA, USA). Significance was accepted at *p* < 0.05. Linear relationships were explored by Pearson's correlation.

## 3. Results

Table 1 shows the plasma concentration of CRP and cytokines before and immediately after a street dance class. Although we did not find differences in the plasma concentration of CRP, TNF-*α*, IL-6, IL-10, and IL-8 (Table 1), the street dance class resulted in an increase in plasma IL-1*β* levels (4.06%, *p* < 0.005) (Table 1).

**Figure 1:**
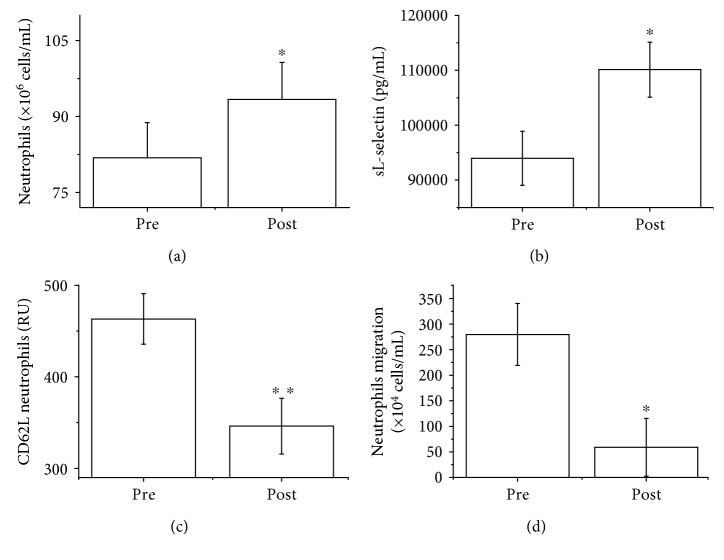
Total count of neutrophils (×10^6^ cells/mL) (a), plasma sL-selectin (pg/mL) (b), CD62L expression (relative units (RU)) (c), and migration (×10^6^ cells/mL) (d) of neutrophils determined before and immediately after the street dance class. The values are presented as the mean ± SE for 10–14 dancers. ^∗^*p* < 0.05 and ^∗∗^*p* < 0.01 (compared with the before-class condition).

As shown in Figure 1, the street dance class resulted in an increase in total neutrophil count (12.36%, *p* < 0.05) and in the concentration of sL-selectin (17.67%, *p* < 0.05) (Figures 1(a) and 1(b), resp.). On the other hand, the street dance class resulted in a reduction in neutrophil CD62L (25.27%, *p* < 0.01) and neutrophil migration (78.92%, *p* < 0.05) (Figures 1(c) and 1(d), resp.).

**Figure 2:**
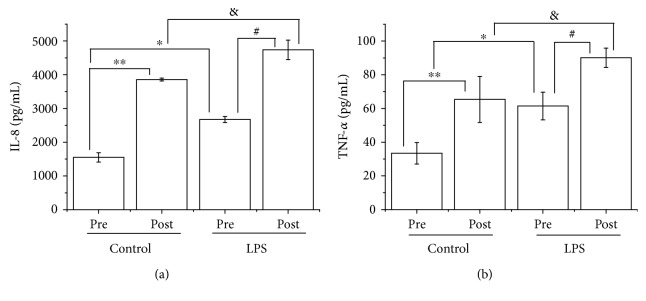
IL-8 (pg/mL) (a) and TNF-*α* (pg/mL) (b) release by neutrophils before and immediately after a street dance class. The measurements were taken in the control and LPS-stimulated conditions. The values are presented as mean ± SE of 9–14 players. ^∗^*p* < 0.05 compared with the before-class condition (no LPS); ^∗∗^*p* < 0.01 compared with the before-class condition (no LPS); ^#^*p* < 0.05 compared with the before-class condition (LPS); ^&^*p* < 0.05 compared with the postclass condition (no LPS).

LPS stimulation resulted in an increase in neutrophil production of IL-8 (41.95% (baseline) and 18.59 (post), *p* < 0.05) and TNF-*α* (45.9% (baseline) and 27.77 (post), *p* < 0.05) (Figures 2(a) and 2(b), resp.). Moreover, neutrophil production of IL-8 (Figure 2(a)) and TNF-*α* (Figure 2(b)) increased by 59.75% (*p* < 0.01) and 49.23% (*p* < 0.01), respectively, in the basal condition, and by 43.55% (*p* < 0.05) and 32.22% (*p* < 0.05), respectively, in LPS-stimulated conditions, after the street dance class.

**Figure 3:**
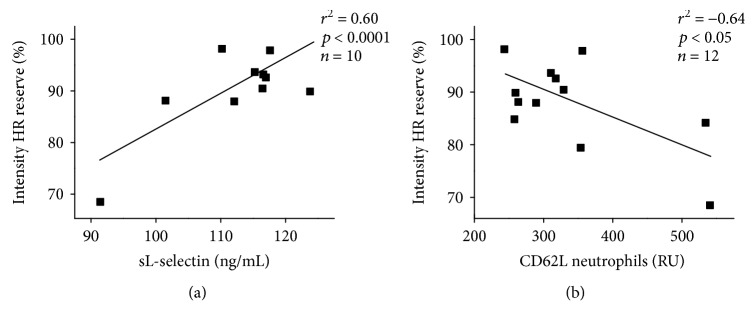
The degree of linear relationship between exercise intensity and sL-selectin plasma levels (*r*^2^ = 0.60, *p* < 0.0001) (a) and with the expression of CD62L by neutrophils (*r*^2^ = −0.64, *p* < 0.05) (b) was established by Pearson's correlation. Levels of sL-selectin and expression of CD62L were quantified before and immediately after the street dance class. Values refer to absolute post values and are presented as the means ± SE for 8–12 dancers in terms of nanograms per milliliter (ng/mL) and RU, respectively. ^∗^*p* < 0.01 compared with the before-class condition.

Figure 3 shows a positive correlation between plasma sL-selectin concentration and the intensity of dancing performed, measured via HR reserve (*r*^2^ = 0.60, *p* < 0.0001) (Figure 3(a)), and a negative correlation between the neutrophil expression of CD62L and the intensity of dancing (*r*^2^ = −0.64, *p* < 0.05) (Figure 3(b)).

## 4. Discussion

Our results demonstrated that a single session of street dancing, lasting 60 minutes, increased the concentration of IL-1*β* and sL-selectin, indicating the induction of inflammation. sL-selectin, from neutrophils, is found in the plasma at high levels, and it may retain functional activity, indicating a possible role for sL-selectin in the regulation of leukocyte attachment to endothelium [14]. sL-selectin also plays an important role in the migration of lymphocytes into peripheral lymph nodes and sites of chronic inflammation and in the migration of neutrophils to acute inflammatory sites [8]. Interestingly, our results showed a positive correlation between sL-selectin and dance intensity. Since selectins have been reported to promote the subsequent activation of immune cells, playing a critical role for the initiation of leukocyte responses [9], our data indicate that increasing dance intensity could result in greater inflammation and increases in plasma soluble adhesion molecules.

After the class, neutrophil count increased but the expression of CD62L on neutrophils was lower. In addition, we found that neutrophil productions of IL-8 and TNF-*α* in the control and LPS-stimulated conditions were increased and there was a reduction in neutrophil migration immediately after the dance class. In contrast to our data, some investigations have reported increased neutrophil chemotactic activity after moderate exercise [15, 16]. However, the intensity, type and duration of exercise can influence leukocyte immune responses [17, 18], and it should be noted that the dance class in this study was performed at an intensity of 85–90% of the HR reserve, which represents a high-intensity activity. Moreover, the mechanism for the decreased chemotactic activity in athletes may be related to humoral factors which influence chemotaxis or may imply exercise-induced intrinsic alterations in the cell itself [19]. In order to investigate further impaired chemotaxis after submaximal aerobic exercise, Gavrieli et al. [20] studied adult males before and 24 hours after 30 minutes of treadmill running at 75% of maximal oxygen uptake (VO_2_ max). Similar to our data, they found that neutrophil chemotaxis was impaired after exercise [20]. They suggested that the pathophysiological mechanism of impaired migration is likely associated with impaired postexercise neutrophil adherence [20], a hypothesis supported by previous studies that have demonstrated that submaximal exercise reduces leukocyte adherence in active men [21, 22].

Although the clinical significance of the transient reduction in neutrophil chemotaxis after intense exercise, such as street dance, is still not clear, it is important to keep in mind that neutrophil function is a key component in the resolution of infection and the control of inflammation [8]. Once in the lung parenchyma, for example, neutrophils release antimicrobial factors and phagocytose bacteria that reduce the action of pathogens. Nevertheless, these mediators can also deteriorate host tissue if the neutrophil-mediated inflammatory response is not properly controlled, resulting in an elevated susceptibility to respiratory infections [23]. Thus, it is likely that the reduction in neutrophil chemotaxis and increased inflammation status of dancers reflects an “open window” in which host defense is reduced and the risk of infection is elevated [17]. It is also important to mention that, prior to this study, research on street dance and inflammation has been primarily descriptive or restricted to case studies and further long-term studies are needed to confirm whether street dancers do have higher infection prevalence/incidence.

In fact, short-term, high-intensity exercise can lead to a significant and prolonged dysfunction of the peripheral blood leukocytes, which is accompanied by an increase in proinflammatory mediators. To illustrate this, Tuan et al. [24] investigated 12 healthy volunteers for 3 consecutive days of high-intensity exercise training (85% of VO_2_ max for 30 minutes every day). Plasma concentrations of TNF-*α* and soluble Fas ligand were raised during the exercise sessions and had not returned to normal 72 hours after the completion of exercise [24]. This supports the potentially deleterious effects of excessive exercise on immune function and health [18].

The current study found that neutrophil numbers increased immediately following a street dance class. However, the expression of CD62L by neutrophils was reduced by the class. We observed a negative correlation between exercise intensity and expression of CD62L, indicating that effect of exercise on the expression of adhesion molecules, an important event for the rolling, adhesion, and migration of neutrophils [9], is intensity dependent. Previous studies have already demonstrated the role of CD62L adhesion molecules in the transmigration of leukocytes, performing critical points of the immune role of host defense [25].

As expected, the neutrophil production of IL-8 and TNF-*α* was higher with LPS stimulation. An important aspect of neutrophil function is their ability to modulate the inflammatory response via the systemic production of cytokines, such as TNF-*α* and other inflammatory mediators [26]. These leukocytes have a high phagocytic capacity and, when a pathogen is engulfed, releases IL-8, which recruits further neutrophils to the site of infection [27]. Moreover, the street dance class increased the basal responsiveness of neutrophils, increasing the release of TNF-*α* and IL-8 in the nonstimulated (absence of stimuli) and stimulated conditions. This increased production of cytokines by neutrophils after the dance class points to dancing as a priming agent for subclinical inflammation.

The serum concentrations of TNF-*α*, IL-1*β*, and IL-8 are associated with the severity of inflammatory processes [6]. Also, the plasma increase in IL-1*β* in the current study agrees with the current literature, as other studies have shown that intense exercise [7], and even some dance modalities [4], induces muscle injury and, as a consequence, leads to increase in proinflammatory markers [10, 28]. Moreover, Keller et al. [29] demonstrated a marked increase in plasma levels of some cytokines not seen in our study, such as IL-6 [29]; however, local tissue IL-6 elevation is noted mostly during shorter exercise, whereas systemic IL-6 increases are usually observed only with longer exercise [30, 31]. The current exercise protocol had a short time duration (60 minutes), and even if there was a local tissue increase of IL-6, this was not evident in the systemic circulation. Thus, studies that target local tissue levels of cytokines related to the effect of dance could represent a promising path in future research.

Intensive exercise suppresses several leukocyte functions including chemotaxis and the release of cytokines by leukocytes [32]. The findings of this study support the concept that intense street dance classes may adversely affect the immune system, demonstrating the potential for street dance to negatively modify neutrophil function which will potentially increase the risk of infectious diseases in amateur dancers. Future studies are necessary to assess the risk of infection in dancers over the long term.

## 5. Conclusion

An acute bout of street dancing induces inflammation and reduces neutrophil migration and adhesion molecule expression. These findings improve our understanding of the risk of infection following acute dance exercise, and it may represent a useful tool to design new studies and strategies to prevent a dancer's performance from declining as a result of inflammation/infection.

## Figures and Tables

**Table 1:** Effect of participation in a street dance class on plasma CRP, IL-1*β*, TNF-*α*, IL-6, IL-10, and IL-8 in terms of picograms per milliliter (pg/mL). Values were determined before and immediately after the class and are presented as the means ± SE for 9–13 dancers. ^∗^*p* < 0.005 (compared with the before-class condition).

	Pre	Post
CRP (pg/mL)	1157.63 ± 49.51	1037.11 ± 73.03
IL-1*β* (pg/mL)	8.04 ± 0.08	8.38 ± 0.11^∗^
TNF-*α* (pg/mL)	481.82 ± 118.03	469.07 ± 104.79
IL-6 (pg/mL)	26.73 ± 6.55	26.37 ± 5.23
IL-10 (pg/mL)	73.63 ± 0.59	74.17 ± 0.75
IL-8 (pg/mL)	25.27 ± 0.33	25.43 ± 0.44

## Data Availability

Data arising from this study are contained within the manuscript.
